# Hybrid Palliation for Hypoplastic Borderline Left Ventricle: One More Chance to Biventricular Repair

**DOI:** 10.3390/children10050859

**Published:** 2023-05-11

**Authors:** Lilia Oreto, Giuseppe Mandraffino, Rita Emanuela Calaciura, Daniela Poli, Placido Gitto, Michele Benedetto Saitta, Ermanno Bellanti, Scipione Carerj, Concetta Zito, Fiore Salvatore Iorio, Paolo Guccione, Salvatore Agati

**Affiliations:** 1 Department of Clinical and Experimental Medicine, University of Messina, 98122 Messina, Italy; gmandraffino@unime.it (G.M.); scarerj@unime.it (S.C.); czito@unime.it (C.Z.); 2 Mediterranean Pediatric Cardiology Center, Bambino Gesù Children’s Hospital, 98035 Taormina, Italy; ritacalaciura@gmail.com (R.E.C.); polidany@hotmail.com (D.P.); dgitto72@yahoo.it (P.G.); miki.sait@yahoo.it (M.B.S.); ermannobellanti@icloud.com (E.B.); fsiorio@yahoo.com (F.S.I.); paolo.guccione@opbg.net (P.G.); salvatore.agati@opbg.net (S.A.)

**Keywords:** hybrid palliation, hypoplastic left heart syndrome, echocardiography, borderline left ventricle, biventricular repair

## Abstract

Treatment options for hypoplastic borderline left ventricle (LV) are critically dependent on the development of the LV itself and include different types of univentricular palliation or biventricular repair performed at birth. Since hybrid palliation allows deferring major surgery to 4–6 months, in borderline cases, the decision can be postponed until the LV has expressed its growth potential. We aimed to evaluate anatomic modifications of borderline LV after hybrid palliation. We retrospectively reviewed data from 45 consecutive patients with hypoplastic LV who underwent hybrid palliation at birth between 2011 and 2015. Sixteen patients (mean weight 3.15 Kg) exhibited borderline LV and were considered for potential LV growth. After 5 months, five patients underwent univentricular palliation (Group 1), eight biventricular repairs (Group 2) and three died before surgery. Echocardiograms of Groups 1 and 2 were reviewed, comparing LV structures at birth and after 5 months. Although, at birth, all LV measurements were far below the normal limits, after 5 months, LV mass in Group 2 was almost normal, while in Group 1, no growth was evident. However, aortic root diameter and long axis ratio were significantly higher in Group 2 already at birth. Hybrid palliation can be positively considered as a “bridge-to-decision” for borderline LV. Echocardiography plays a key role in monitoring the growth of borderline LV.

## 1. Introduction

Hypoplastic left heart syndrome (HLHS) encompasses a spectrum of diseases sharing the common feature of an underdeveloped left ventricle (LV), which is inadequate to support systemic circulation. Pathological entities of HLHS are basically mitral atresia or stenosis, aortic atresia or stenosis, and hypoplastic or interrupted aortic arch, variably combined with each other and with variable degrees of expression [1,2,3]. Treatment options for HLHS are critically dependent on the degree of development of the left ventricle, whatever the anatomical variant [4]. Severely hypoplastic LV undergoes single ventricle-staged palliation, whereas mild hypoplasia allows the LV to deal with the systemic circulation. Yet, there is a consistent number of cases where LV lies just in between the two described situations, so the choice of the appropriate treatment soon after birth can be challenging. Several parameters of LV adequacy are available by echocardiography and can help to orient between the two pathways [5,6]. However, it is now clear that the timing of the decision plays a crucial role in the fate of the hypoplastic borderline LV because the longer the ventricle is allowed to grow, the higher the probability of achieving functional independence. In this setting, the advent of hybrid palliation for HLHS has gained favor since it is conceived to artificially maintain fetal circulation for some months, thus deferring major surgery far beyond neonatal term [7,8]. Hybrid stage I palliation is a less invasive alternative to classical Norwood stage I operation, based on a combination of a surgical and an interventional approach. Via median sternotomy, surgical banding of the pulmonary artery branches is followed by transcatheter stenting of the ductus arteriosus by direct puncture of the main pulmonary trunk. Therefore, the main goals of the Norwood operation, which means unobstructed systemic outflow (with aortic arch reconstruction and aorto-pulmonary amalgamation) and protected pulmonary flow (by a systemic-to-pulmonary shunt), are here replaced by the ductal stent and the selective pulmonary arteries banding, respectively. Such a procedure does not require cardiopulmonary bypass and circulatory arrest and is clearly less invasive for the newborn patient compared to the Norwood operation.

Being a temporary palliation, in the case of borderline LV, the goal is to postpone the decision until the time the LV has fully expressed its growth potential, possibly increasing the number of biventricular repairs [9,10].

The aim of this study was to describe the growth potential of hypoplastic borderline LV in patients who underwent hybrid stage I palliation with banding of pulmonary artery branches and stenting of the ductus arteriosus [9,11,12,13]. Therefore, we focused our attention on those patients who presented with any kind of borderline hypoplastic left heart, aiming to identify echocardiographic parameters that can help evaluate LV growth from birth to surgical stage II.

## 2. Materials and Methods

**Figure 1:**
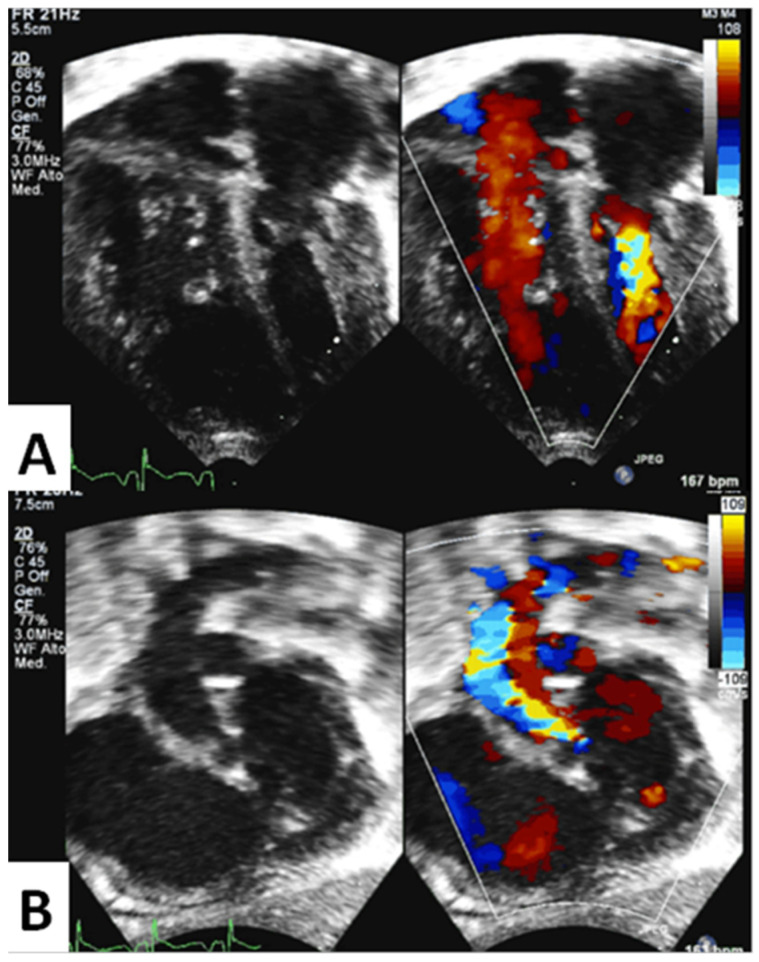
Panel (**A**). Apical 4−chamber view of a severely underdeveloped left ventricle with mitral annular hypoplasia. Panel (**B**). Subcostal left oblique view showing severe aortic annular and arch hypoplasia; aortic stenosis is evident with color−Doppler. Antegrade aortic flow is obstructed but still present.

Patients. We retrospectively reviewed clinical and echocardiographic data of 45 patients born with different HLHS variants who underwent hybrid palliation with surgical selective banding of pulmonary artery branches and transcatheter stenting of the ductus arteriosus at our Institution between October 2011 and January 2015. The operation was performed as a single procedure, via median sternotomy, with no need for cardiopulmonary bypass and circulatory arrest. Informed consent was obtained for each patient according to our institutional policy. A necessary condition for potential LV growth is a patent LV inflow and outflow with a preserved antegrade flow. Therefore, we reviewed our population searching for these primary features. We did not expect any growth in the worst end of the spectrum, i.e., mitral atresia/aortic atresia (MA/AA) or mitral stenosis/aortic atresia (MS/AA) variants (22 patients). Likewise, we did not include mildly hypoplastic LV as in aortic arch hypoplasia/interruption with ventricular septal defect (7 patients), in which biventricular repair is affordable even with critically small LV volumes. In between the mild and the severe HLHS variants, our group with intermediate features included 13 patients with mitral and aortic hypoplasia (less than −2 z score) and 3 patients with unbalanced atrio-ventricular septal defect (uAVSD). All 16 patients showed a patent LV inflow and outflow and therefore were considered for a potential left heart growth. Their main characteristics were (a) antegrade aortic flow and (b) severely underdeveloped LV, expressed by LV mass below −2 Z scores (Figure 1).

**Figure 2:**
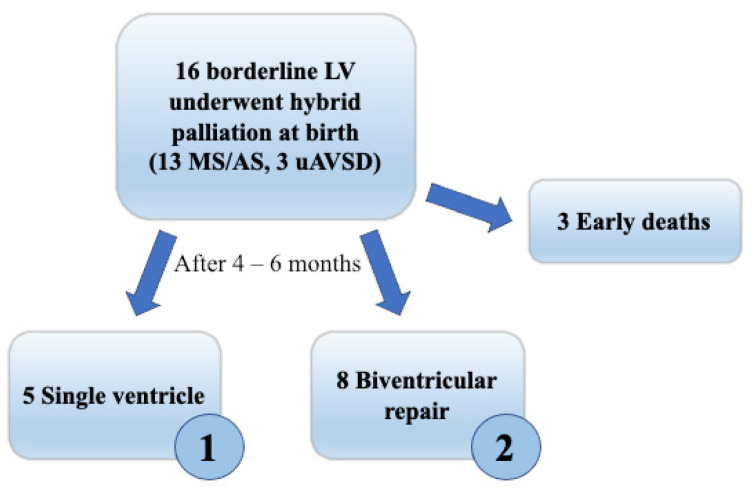
Study design. Among all patients with hypoplastic borderline left ventricle, we distinguished 2 groups according to the subsequent surgical strategy they underwent after interstage: Group 1, univentricular palliation; Group 2 biventricular repair (Legend: LV, left ventricle; MS, mitral stenosis; AS, aortic stenosis; uAVSD, unbalanced atrioventricular septal defect).

According to the surgical treatment received after an interstage period of 4–6 months, this borderline group was further subdivided (Figure 2): Group 1 had single ventricle staged palliation (5 patients), Group 2 had biventricular repair (8 patients), while 3 patients died before surgery, and one is currently on interstage follow-up. Patients in Group 2 underwent ventricular septal defect closure and aortic arch reconstruction, with the creation of a calibrated atrial septal defect, to allow gradual adaptation of the LV to augmented preload.

**Figure 3:**
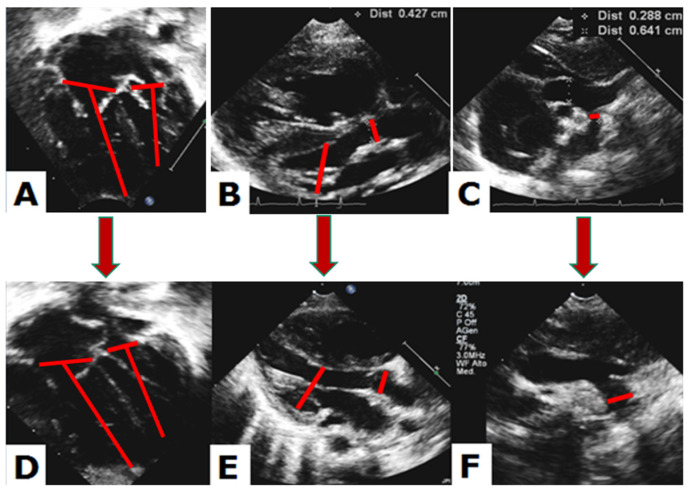
Case example from Group 2. Left heart structures are shown at birth (superior panels) and after interstage (inferior panels). Mitral and tricuspid annulus, and long axis of both ventricles (**A**,**D**), aortic root and end-diastolic left ventricular diameter (**B**,**E**), and distal aortic arch diameter (**C**,**F**) are depicted in red.

Echocardiography. Two-dimensional echocardiograms of patients from Groups 1 and 2 were retrospectively reviewed, comparing examinations recorded at birth with those obtained 5 months after hybrid palliation. Quantitative analysis included measurement of aortic annulus, aortic root, mitral and tricuspid annulus, LV systolic and diastolic diameters and volumes, LV mass, LV long-axis to heart long-axis ratio (LAR), transverse arch, distal arch and aortic isthmus diameters, according to the American Society of Echocardiography recommendations [14]. Each measurement was then expressed as an indexed value (by DuBois formula for body surface area) or reported as z-score. The aortic annulus and root were measured in mesosystole from the parasternal long-axis view with inner-to-inner edge method. From the apical 4-chamber view were obtained mitral and tricuspid annulus in protodiastole, systolic and diastolic LV volumes and LAR. In particular, LAR was calculated as the ratio of LV long axis from the mitral plane to the endocardial border, divided by the heart long axis from the tricuspid plane to the epicardial border of the right ventricle (Figure 3) [15]. In the case of AVSD, annular planes and ventricular volumes were measured by tracing an imaginary line of the crux cordis from the edge of the ventricular septum [16]. LV mass was calculated by the formula

0.8{1.04[([LVEDD + IVSd + PWd]3 − LVEDD3)]} + 0.6

Using LV end-diastolic diameter (LVEDD), ventricular septal thickness (IVSd) and LV posterior wall (PWd) were measured at end-diastole from the parasternal long-axis view. Transverse and distal arch and aortic isthmus diameters were measured at end-systole, i.e., at the maximum expansion of the vessel, between the first two epi-aortic branches, between the second and the third and below the third branch, respectively. Furthermore, we evaluated anatomical features such as endocardial fibroelastosis, bicuspid aortic valve, single papillary muscle of the mitral valve, persistent left superior vena cava and small inter-atrial communication in order to examine if any of these qualitative characteristics could influence the growth potential of the LV.

Finally, we applied the original “Rhodes score” and the “Threshold score” reported by Rhodes et al. [17] and the revised “Discriminant score” described by Colan et al. [15] to all the patients at birth in order to test the potential predictive value of the existing scores in this population with multi-level left heart obstruction, even though the same scores were created for critical aortic stenosis. Requested parameters include weight and height, aortic annulus (mm), aortic root (mm), mitral annulus (mm) measured in apical four-chamber and parasternal long axis views, LV long axis and heart long axis (mm), and grade of endocardial fibroelastosis (0–3). For the Rhodes criteria, a score of less than −0.35 was predictive of death after two-ventricle repair; a Threshold score of 2 or more suggests that high mortality after two-ventricle repair; a Discriminant score with a cutoff value of -0.65 predicted outcome in 95% of survivors and 80% of events (90% overall).

Follow-up. We collected data about the subsequent clinical and surgical history of patients with borderline LV until 2022.

Statistical Analysis. A non-parametric, permutation-based analysis was performed, considering the small sample size [18]; indeed, this approach allows for a deeper exploration of the data with respect to a classical non-parametric approach, especially in smaller sample sizes. The NPC-Test (http://static.gest.unipd.it/~salmaso/NPC_TEST.htm (accessed on 18 March 2023)) was used to estimate the mean difference between the groups, and within the groups for repeated measures; consistently, data are presented as mean ± standard deviation. Further, we estimated the probability of obtaining the assignment to Groups 1 (single ventricle palliation) or 2 (biventricular repair) through the associations between this one and the other dichotomous variables (endocardial fibroelastosis; restrictive atrial communication). A logistic multinomial model was estimated in order to verify if any continuous variable was able to predict the probability of being assigned to Groups 1 or 2. SPSS statistical package version 26.0 (Chicago, IL, USA) was used to perform the analyses.

## 3. Results

Characteristics of patients in the two groups were homogeneous in terms of anthropometric parameters, anatomical variants and age at intervention and are reported in Table 1.

### 3.1. Quantitative Echocardiographic Evaluation

Echocardiographic measurements for both groups at birth and after 5-month follow-up are reported in Table 2 and include LAR, Z score of the aortic annulus, aortic root, aortic arch, mitral annulus and LV mass, indexed measures of the aortic root, LV mass and systolic and diastolic LV volumes.

At birth, hypoplasia of the LV was equally severe in the two groups, with an LV mass z score of −5.58 vs. −4.89 (Group 1 vs. Group 2), small, indexed volumes (7.44 vs. 10.08 mL/m^2^) and mitral annulus (−4.21 vs. −4.13).

**Figure 4:**
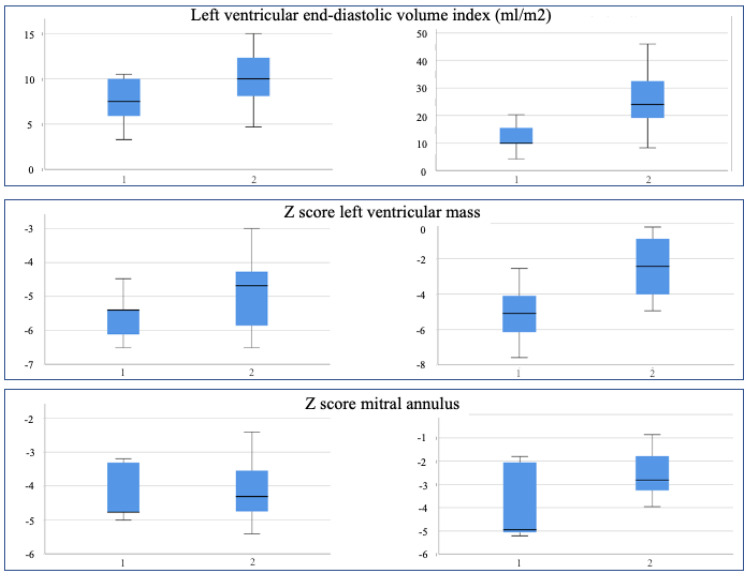
Graphical representation of different parameters in Group 1 (single ventricle palliation) and Group 2 (biventricular repair) at birth (**left panels**) and after 5 months (**right panels**).

Conversely, after a 5-month follow-up, the same parameters have significantly changed in Group 2, particularly LV volumes and LV mass z score (from −4.89 to −2.48), but not in Group 1 (Figure 4).

With regards to the aortic segment, however, a significant difference between the groups was already present at birth since the aortic root z score was −5.13 in Group 1 and −3.31 in Group 2 (*p* 0.053). The same difference was noted for the LAR, measured at 0.67 and 0.79, respectively (*p* 0.054). The aortic annulus z score and indexed aortic root showed a similar trend but did not reach statistical significance (*p* 0.071).

**Figure 5:**
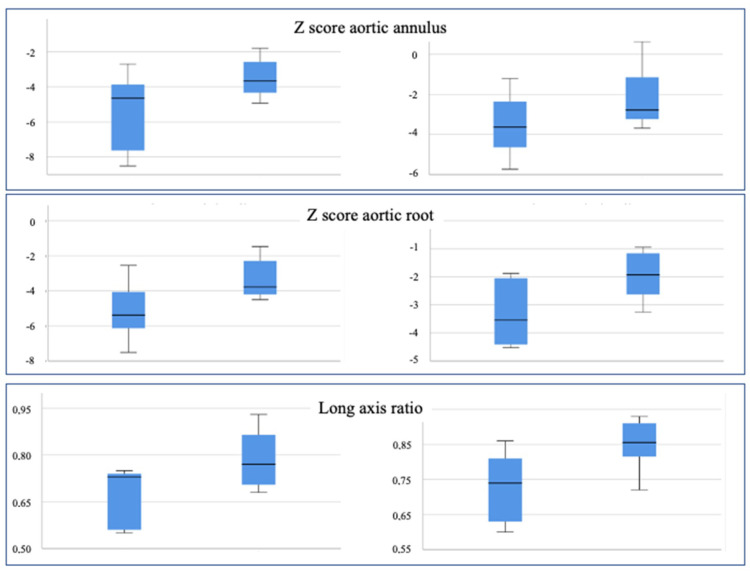
Graphical representation of different parameters in Group 1 (single ventricle palliation) and Group 2 (biventricular repair) at birth (**left panels**) and after 5 months (**right panels**).

Similar results could be observed for the aortic segment and the LAR after a 5-month follow-up since these parameters were somewhat augmented in both groups (Figure 5).

From a different point of view, the same data could be observed separately for each group, focusing on how the same parameters would change over time (Table 3):

In Group 1, aortic annulus and aortic root z scores have increased significantly during follow-up (*p* 0.043 and 0.042, respectively), and the same occurred to the end-diastolic volume (*p* 0.043) and LAR (*p* 0.042). However, none of these parameters reached the normal range, and this explains why patients of Group 1 were assigned to univentricular palliation.

On the other hand, all components of the left heart have grown significantly during follow-up in Group 2: aortic annulus (*p* 0.036), aortic root (*p* 0.017), mitral annulus (*p* 0.012), LV mass (*p* 0.012) and indexed end-diastolic and end-systolic volumes (*p* 0.012 and *p* 0.013). As a result, LV growth was deemed sufficient to reach eligibility for biventricular repair.

As concerns the aortic arch, no significant difference was observed between the two groups, neither at birth nor after follow-up. Likewise, no significant growth of the aortic arch could be observed in any of the two groups over time.

### 3.2. Qualitative Echocardiographic Evaluation

The rate of endocardial fibroelastosis, restrictive atrial communication, bicuspid aortic valve, mitral valve with single papillary muscle and left superior vena cava is reported in Table 1. Of note, endocardial fibroelastosis did not influence the probability of being in Group 1 or 2, while restrictive atrial communication was 4.5 times more frequent in Group 2.

### 3.3. Application of Pre-Existing Risk Scores for Biventricular Repair

The Rhodes score (cutoff −0.35) and the Threshold score (2 or more) suggested univentricular palliation for the entire population of the present study at birth. For all patients bar one, the Discriminant score predicted single ventricle palliation as the preferred treatment option (cutoff −0.65). We obtained the following results:

Rhodes score:

Group 1: −3.9; −4.02; −2.73; −3.56; −2.1

Group 2: −2.39; −1.6; −2.95; −1.23; −2.98; −1.84; −1.76; −2.23

Threshold score:

Group 1: 4, 4, 4, 4, 4

Group 2: 4, 3, 4, 2, 4, 4, 3, 3

Revised Discriminant score:

Group 1: −4.9; −4.2; −2.04; −3.09; −1.58

Group 2: −1.26; −1.23; −1.95; −0.33; −2.41; −1.89; −1.45; −1.59

### 3.4. Follow-Up

Long-term follow-up was available for 12 patients, with a median time of 8 years (range 4–10 years). In Group 1, two patients died (one from endocarditis after stage II and one after Fontan operation); in Group 2, two patients were lost to follow-up. Among our remaining three patients from Group 1, one patient with partial AV canal demonstrated such a significant growth of LV structure by magnetic resonance imaging [19,20] that, after 4 years, she was deemed eligible for conversion towards a biventricular repair; today, after 4 years, she has a good functional capacity, with mild-to-moderate pulmonary hypertension, moderate mitral regurgitation, and is free from subsequent surgery/intervention. The other two patients from Group 1 were not candidates for Fontan operation due to pulmonary hypertension. All six patients from Group 2 are alive and free from subsequent surgery, although three of them required interventional dilatation of pulmonary branches.

## 4. Discussion

Among the benefits of hybrid palliation for HLHS, the role of “bridge-to-decision” is gaining considerable value [10]. In fact, independent of which therapeutic pathway is taken, the hybrid palliation has the advantage of keeping all potential approaches viable, from classic Norwood operation to hybrid stage II palliation, to biventricular repair, all the way up to heart transplant [9,21].

In dealing with a neonate with multiple obstructive lesions of the left heart and borderline LV, the decision between univentricular or biventricular repair is often required in the earlier days of life, and it may not be easily reversible [22]. Consequences of inappropriate selection can be very severe when a potentially biventricular heart is “constrained” to univentricular physiology or a “non-systemic” LV must manage a disproportionate load, with fatal effects in the absence of prompt reintervention. Therefore, the most accurate selection of patients is paramount and is strictly linked to the evaluation of cardiac anatomy based on the two major diagnostic techniques, echocardiography and cardiac magnetic resonance imaging.

Our study showed that 8/13 patients with borderline LV who were not eligible or considered at extremely high risk for biventricular repair at birth became good candidates for two-ventricle physiology 5 months after hybrid palliation.

All of our 13 patients with borderline LV showed similar characteristics and uniformly distributed anatomical variants. Although, at birth, all LV measurements were far below the normal range in both groups, particularly LV mass (−5.5 z score in Group 1, −4.8 z score in Group 2), 5 months after the hybrid procedure, the routes diverged significantly, given that LV mass in Group 2 was fairly close to normal (−2.4 z score), while in Group 1, it did not show any growth (−5.1 z score).

Therefore, based on the homogeneity of the 13 neonates at birth, it would have not been possible to distinguish which patient could reach biventricular repair and which could not. Nonetheless, from a careful analysis of their characteristics at birth, it can be observed that small yet significant differences were already present at that time, which may have raised suspicion about which group a patient could be then assigned to. Aortic root diameter and LAR were significantly higher in Group 2 at birth, and similarly higher was the aortic annulus, though it did not reach statistical significance.

Large-scale studies have been carried out on neonates with critical aortic stenosis to identify quantitative criteria that would be able to guide treatment choice. Some of these criteria are the “Rhodes scores” [17], the apex-non-forming LV, the endomyocardial fibroelastosis, indexed LV end-diastolic volume <20 mL/m^2^, elevated end-diastolic pressure and depressed contractile function of the LV [23]. In particular, the “threshold score” of Rhodes is an ordinal scoring system based on four points: indexed aortic root diameter < 3.5 cm/m^2^; indexed MV area < 4.75 cm^2^/m^2^; LAR < 0.8; indexed LV mass < 35 g/m^2^; a score of two or more means the ventricle is not suitable for biventricular repair [15,17].

Being aware that these scores were created for critical aortic stenosis, we still applied them to our population with multi-level left heart obstruction; we found that the Rhodes score and the Threshold score clearly discouraged any attempt to biventricular repair, while the revised Discriminant score allowed biventricular repair in only one patient. Even if these tools may not be appropriate for our population, on the other hand, their use supports our choice of taking time with hybrid palliation as a bridge-to-decision, given that the majority of our patients achieved a successful biventricular repair after 5 months. A different setting is an aortic coarctation with various degrees of aortic arch hypoplasia. In fact, the criteria formulated for aortic valve stenosis are not applicable for aortic coarctation with arch hypoplasia because even a severely small LV (volume < 10 mL/m^2^), not forming cardiac apex, has a significant growth potential once the obstructive outflow and the right ventricular volume overload have been resolved [23,24,25].

In any case of LV compression from the right ventricular volume overload, as in unbalanced AVSD, cardiac magnetic resonance imaging plays a key role since it is also able to measure the potential LV volume obtained by an algorithm transforming the crescentic shape of the compressed LV into an ellipsoidal shape, as it is expected after the compression has been resolved [26]. The same methodology, although with less accuracy, has been applied to echocardiography for preoperative evaluation of patients with unbalanced AVSD [16,27,28,29,30].

Nevertheless, apart from well-defined entities such as critical aortic stenosis and aortic coarctation with arch hypoplasia, a wide spectrum of anomalies with borderline left ventricle exists, for which the issue is still open [31,32,33]. In this heterogeneous class of neonates, the decision about therapeutic strategy is not supported by reliable quantitative criteria; rather, it is based on expert agreement tailored for each given case. In this context, the added value of hybrid palliation as a “bridge-to-decision” is evident since it allows a temporal window of months, during which a serial and focused evaluation can be carried on for each patient, leading eventually to the most appropriate option.

Although the Threshold score system by Rhodes [15,17] cannot be applied to a population different from isolated critical aortic stenosis, it should be noted that two out of four elements of the score are precisely aortic root diameter and LAR. Indeed, our population is too small to draw referral numbers, but our data are quite similar to those given by the score. It can be speculated that the reason why the other two elements of the score do not apply as well to our population is that mitral valve area and LV mass may not be equally involved in isolated aortic stenosis, as they are in our patients with multiple left heart obstructions.

As opposed to the aortic root, we could not notice any significant growth of the aortic arch at the end of the interstage period. A possible explanation might be that the development of the aortic root, promoted by the increasing LV output, is directly related to the underlying growth of the LV, while the aortic arch laid just in between the antegrade and retrograde flow, which may not support adequately its expansion.

Associated anatomical features were evaluated in order to clarify their possible influence on LV growth. As we expected, atrial communication was relatively smaller in the biventricular group, according to the hypothesis that an augmented pre-load could promote LV development. In fact, restrictive atrial septal defect, promoting LV filling, was 4.5 more common in Group 2. In this regard, surgical restriction of atrial communication is one of the main approaches used for LV recruitment [19].

Conversely, endocardial fibroelastosis did not appear to influence the probability of having a single ventricle physiology; however, ultrasound evaluation of endocardial fibroelastosis is consistently limited compared to magnetic resonance [34]. We may speculate that our evaluation of endocardial fibroelastosis based on the qualitative expression on echocardiography is not adequate to discriminate the presence or absence of potential for LV growth. The amount of endocardial fibroelastosis has a strong impact on the diastolic properties of the LV; thus, it has an influence on postcapillary pressure. In our population, it is not easy to assess the actual impact of postcapillary hypertension since its variable degree has to be added to a certain grade of pulmonary artery distortion, as accounted by a not infrequent need for interventional repeated pulmonary branch dilatation. The bicuspid aortic valve appeared to be a little more common in the biventricular group, although not significantly. Surprisingly, single papillary muscle and persistent left superior vena cava, which could potentially discourage LV development, are both relatively common in our biventricular group, while they are absent in Group 1.

A significant limitation of this study is that the number of patients is dramatically small due to the relatively rare prevalence of the disease and the monocentric nature of the study. For the same reason, there are no reference values available for this category of patients, as there are for isolated aortic stenosis and other more common pathologies.

Nevertheless, the benefits of hybrid palliation as a bridge-to-decision for the borderline LV are observed by echocardiographic demonstration of LV growth during interstage. Future studies could test the hypothesis that a watchful observation of critical targets at birth could be useful to guide the heart team on the appropriate, early choice between univentricular palliation and biventricular repair.

## 5. Conclusions

Hybrid palliation for HLHS offers a number of benefits; one of them is to act as a “bridge-to-decision” in case of borderline LV. In fact, the goal is to postpone the decision until the time the LV has fully expressed its growth potential in order to increase the possibility to achieve a biventricular repair. Echocardiography has a key role in identifying borderline LV, monitoring its modification over time and thus orienting toward the most appropriate therapeutic approach. For instance, the choice is based on careful measurements of the left heart structures at the end of the interstage, but it is equally important to evaluate their growth potential compared to what they appeared at birth. Finally, it might be useful to identify some clues, already at birth, that can predict the fate of the borderline LV thereafter, but this requires further detailed studies.

## Figures and Tables

**Table 1:** Patients’ characteristics.

	Group 1	Group 2	
Gender	1 M–4 F	4 M–4 F	
Weight at birth	3.03 ± 0.54 Kg	3.25 ± 0.39 Kg	p 0.42
Body surface area (BSA) at birth	0.19 ± 0.01	0.20 ± 0.01	p 0.61
Weight at 5 months	5.58 ± 1.1 Kg	4.94 ± 0.64 Kg	p 0.20 p 0.11 OR 1.1
BSA at 5 months	0.31 ± 0.04	0.28 ± 0.03	
Age at first intervention	2.3 days	2.3 days	
Mitral/aortic stenosis	4	7	
Unbalanced AV canal	1	1	
Endocardial fibroelastosis (EFE)	3/5	5/8	
Bicuspid aortic valve	2/5	5/8	
Single papillary muscle	0/5	4/8	OR 4.5
Left superior vena cava	0/5	5/8	
Restrictive atrial septal defect	2/5	5/8	
Death during long-term follow up	2/5	1/8	

**Table 2:** Mean values of echocardiographic parameters are compared between Groups 1 and 2 at birth and after interstage. Abbreviations: LV, left ventricular; EDV, end-diastolic volume; ESV, end-systolic volume. Non-significant differences are highlighted in green, while significant differences are in purple. Light purple indicates a *p* value approaching the level of significance but still non-significant.

Parameter	Group 1 at Birth	Group 2 at Birth	*p*-Value	Group 1 after 5 Months	Group 2 after 5 Months	*p*-Value
Aortic Annulus (z score)	−5.47	−3.49	0.071	−3.52	−2.17	0.169
Aortic Root (z score)	−5.13	−3.31	0.053	−3.28	−1.65	0.06
Aortic Root (mm/m^2^)	2.79	3.33	0.071	2.61	3.31	0.041
Long axis ratio	0.67	0.79	0.054	0.73	0.85	0.032
Mitral Annulus (z score)	−4.21	−4.13	0.880	−3.81	−2.57	0.132
LV Mass (z score)	−5.58	−4.89	0.271	−5.10	−2.48	0.032
LV Mass (g/m^2^)	20.20	24.00	0.260	29.00	45.38	0.068
Indexed EDV (ml/m^2^)	7.44	10.08	0.168	11.94	25.69	0.034
Indexed ESV (ml/m^2^)	4.00	3.96	0.970	5.20	11.78	0.023
Transverse Arch (z score)	−4.79	−5.44	0.473	−4.17	−4.08	0.944
Distal Arch (z score)	−4.20	−4.70	0.646	−2.55	−3.86	0.403
Aortic Isthmus (z score)	−4.11	−5.41	0.273	−3.08	−3.80	0.570

**Table 3:** Mean values of echocardiographic parameters are measured at birth and after interstage and compared in both Groups. Abbreviations: LV, left ventricular; EDV, end-diastolic volume; ESV, end-systolic volume. Significant differences are highlighted in purple.

Parameter	Group 1 at Birth	Group 1 after 4 Months	*p*-Value	Group 2 at Birth	Group 2 after 4 Months	*p*-Value
Aortic Annulus (z score)	−5.47	−3.52	0.043	−3.49	−2.17	0.036
Aortic Root (z score)	−5.13	−3.28	0.042	−3.31	−1.65	0.017
Long axis ratio	0.67	0.73	0.042	0.79	0.85	0.207
Mitral Annulus (z score)	−4.21	−3.81	0.502	−4.13	−2.57	0.012
LV Mass (z score)	−5.58	−5.10	0.345	−4.89	−2.48	0.012
LV Mass (g/m^2^)	20,20	29.00	0.078	24,00	45.38	0.012
Indexed EDV (ml/m^2^)	7.44	11.94	0.043	10,08	25.69	0.012
Indexed ESV (ml/m^2^)	4.00	5.20	0.08	3.96	11.78	0.013

## Data Availability

The data presented in this study are available on request from the corresponding author. The data are not publicly available due to privacy reason.
